# WHIRLY protein functions in plants

**DOI:** 10.1002/fes3.379

**Published:** 2022-03-17

**Authors:** Rachel E. Taylor, Christopher E. West, Christine H. Foyer

**Affiliations:** ^1^ Faculty of Biological Sciences The Centre for Plant Sciences University of Leeds Leeds UK; ^2^ School of Biosciences College of Life and Environmental Sciences University of Birmingham Birmingham UK

**Keywords:** chloroplasts, DNA damage, homologous recombination, mitochondria, nucleus, pathogenesis‐related genes

## Abstract

Environmental stresses pose a significant threat to food security. Understanding the function of proteins that regulate plant responses to biotic and abiotic stresses is therefore pivotal in developing strategies for crop improvement. The WHIRLY (WHY) family of DNA‐binding proteins are important in this regard because they fulfil a portfolio of important functions in organelles and nuclei. The WHY1 and WHY2 proteins function as transcription factors in the nucleus regulating phytohormone synthesis and associated growth and stress responses, as well as fulfilling crucial roles in DNA and RNA metabolism in plastids and mitochondria. WHY1, WHY2 (and WHY3 proteins in Arabidopsis) maintain organelle genome stability and serve as auxiliary factors for homologous recombination and double‐strand break repair. Our understanding of WHY protein functions has greatly increased in recent years, as has our knowledge of the flexibility of their localization and overlap of functions but there is no review of the topic in the literature. Our aim in this review was therefore to provide a comprehensive overview of the topic, discussing WHY protein functions in nuclei and organelles and highlighting roles in plant development and stress responses. In particular, we consider areas of uncertainty such as the flexible localization of WHY proteins in terms of retrograde signalling connecting mitochondria, plastids, and the nucleus. Moreover, we identify WHY proteins as important targets in plant breeding programmes designed to increase stress tolerance and the sustainability of crop yield in a changing climate.

## Graphical abstract

WHY proteins serve important roles in plant development and stress tolerance. They function as transcription factors in the nucleus, as well as fulfilling crucial roles in DNA and RNA metabolism in chloroplasts and mitochondria. They are excellent potential targets for plant breeding strategies aiming to improve sustainable agriculture.

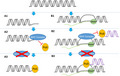

## 1 INTRODUCTION

A rapidly growing, more urbanized population may require a doubling of crop production by 2050 (Tilman et al., 2011). Estimates suggest that maize and wheat production must increase by between 60% and 110% by 2050 to meet increasing demands for food (Daryanto et al., 2016). However, gains in crop productivity made during the green revolution are slowing, with many traits having diminishing scope for improvement (Long et al., 2015). Abiotic environmental stresses such as salinity and drought, as well as biotic stresses such as herbivores and pathogens are major constraints to global food security because they negatively impact plant growth and crop productivity. These environmental stresses currently have negative impacts on the growth and yield of major crops such as wheat and barley (Kong et al., 2020). In wheat and maize up to 40% of crop yield losses are due to drought alone (Daryanto et al., 2016).

Moreover, climate change is predicted to increase the frequency, intensity and duration of extreme weather events that will have further negative impacts on crop production (Ray et al., 2019). Through evolution, plants have acquired highly sophisticated systems that counteract the negative impacts of environmental stresses. Our understanding of the signalling systems, genetic and epigenetic pathways and proteins that regulate plant stress responses stress has greatly increased in recent years (Ku et al., 2018; Lamers et al., 2020). Members of the WHY family of proteins are found throughout the plant kingdom and are predicted to share the ability to bind to single‐stranded DNA to modulate growth and defence responses in the chloroplasts, mitochondria and the nucleus. Our knowledge of WHY functions has considerably increased in recent years (Table 1), confirming that these proteins serve a diverse range of important functions in plant development and stress tolerance. In particular, WHY1 acts as a transcription factor in the nucleus, regulating the expression of large number of genes, which encode housekeeping proteins as well as proteins that regulate plant development and responses to biotic and abiotic stresses. However, the literature on the WHY protein functions can be extremely confusing because there is little overlap in the growth conditions, species and treatments between studies, providing little consensus regarding WHY protein localization and functions across species. Here, we critically analyse the evidence and recent progress in our understanding of the roles of WHY family proteins in the regulation of plant development and responses to abiotic and biotic stresses. We discuss how WHY proteins fulfil a divergent range of key functions in plants, with a consideration of how our growing knowledge of the multifaceted roles of WHY proteins could be used in strategies for sustainable crop improvement.

**TABLE 1:** Recent developments concerning WHIRLY proteins functions

Key findings	Species	Protein	Reference
SlWHY1 was expressed more widely than SlWHY2. Drought and salt stress enhanced levels of SlWHY1 and SlWHY2 transcripts	*Solanum lycopersicum*	WHY1 WHY2	Akbudak and Filiz (2019)
OsWHY1 and OsWHY2 have the highest coverage of proteins bound to OsPAL2;3, an allelopathy promoter. OsWHYs negatively regulate OsPAL2;3	*Oryza sativa*	WHY1 WHY2	Fang et al. (2019)
The MMEJ pathway was blocked by DNA polymerases in the presence of AtWHY2 (and other ssDNA‐binding proteins) in templates of single‐stranded regions longer than 12 nts	*Arabidopsis thaliana*	WHY1 WHY2 WHY3	García‐Medel et al. (2019)
WHY transcripts were decreased in shoots up to 12 h after phytotoxic citral treatment to roots. WHY proteins showed strong affinity for citral isomer binding and low in silico molecular docking	*A. thaliana*	WHY1 WHY2 WHY3	Graña et al. (2020)
SlWHY1 expression was increased by chilling. SlWHY1 acts as a positive regulator of *SlpsbA*, which enhanced chloroplast D1 synthesis. *SlAMY3*‐*L*, a starch‐degrading enzyme, and inhibitor of *SlISA2* starch synthesis‐related enzyme was also regulated by SlWHY1	*Solanum lycospericum*	WHY1	Zhuang, Gao, et al. (2020) and Zhuang, Wang, et al. (2020)
One of the two putative Sorghum WHY TFs that is crucial for pollen development is orthologous to AtWHY2	*Sorghum bicolor*	WHY2	Dhaka et al. (2020)
Plastid genome instability and increased accumulation of ROS were observed in *Atreca1why1why3* mutants, which had leaf growth defects, white variegated sectors, higher accumulations of plastid DNA rearrangements, and reduced fertility	*A. thaliana*	WHY1 WHY3	Duan et al. (2020)
Seed germination was reduced in *Atwhy2* mutants that had an altered mitochondrial structure, disordered nucleoids and increased AtWhy3 levels compared to the wild type. WHY3 was dual targeted to the chloroplasts and mitochondria in protein transport in organello experiments	*A. thaliana*	WHY2 WHY3	Golin et al. (2020)
WHY2 was localized in the mitochondria, plastids and nucleus during leaf ageing. The chloroplasts of pericarp cells of AtWHY2 OE lines had increased starch granule numbers and jasmonate‐associated gene expression linked to early senescence. The opposite phenotype was observed in the *Atwhy2* mutants	*A. thaliana*	WHY2	Huang et al. (2020)
The *Atwhy1* mutants showed early senescence with an early peak in salicylic acid (SA) levels. This was prevented by nuclear WHY1 (nWHY1) expression. Plastid WHY1 (pWHY1) expression enhanced SA levels. The levels of stress‐related transcripts were changed in pWHY1 lines. In contrast, transcripts associated with plant development and early growth were changed in the nWHY1 lines	*A. thaliana*	WHY1	Lin et al. (2020)
SlWHY2 RNAi lines showed a severe wilting phenotype under drought with decreased fresh weight, chlorophyll contents and photosynthesis, as well as decreased expression of mitochondrial DNA repair and recombination genes. ROS accumulation was increased in the SlWHY2 RNAi lines compared to the wild type	*S. lycospericum*	WHY2	Meng et al. (2020)
Weak interactions were observed between CsWHY1 and the highly expressed Irregular Vasculature Patterning (CsIVP). CsIVP targets developmental regulators and functions in downy mildew resistance	*Cucumis sativus*	WHY1	Yan, Ning, et al. (2020)
The expression of MeWHYs and MeCIPK23 was significantly increased 10–20 days of drought. Plants lacking in any or all MeWHYs and/ or MeCIPK23 were more sensitive to drought stress	*Manihot esculenta*	WHY1 WHY2 WHY3	Yan, Liu, et al. (2020)
SlWHY1 OE lines showed reduced wilting under heat stress, with increased levels of SlHSP21.5A transcripts, greater membrane stability and higher soluble sugar contents. ROS levels were decreased relative to the wild type. RNAi lines lacking SlWHY1 showed the opposite phenotype	*S. lycospericum*	WHY1	Zhuang, Gao, et al. (2020)
SlWHY1 was a positive regulator of RuBISCO expression under cold stress directly binding to the promoter of the rbcS gene that encodes the small subunit	*S. lycospericum*	WHY1	Zhuang, Wang, et al. (2020)
WHY2 was shown to be a major regulator of the root apical meristem developmental network	*A. thaliana*	WHY2	McCoy et al. (2021)
Recruitment of the WHY1 and 3 proteins, and AtRNH1C to the same genomic site promoted homologous recombination repair. These proteins maintain chloroplast genome integrity through AtRECA1 interaction. Deletion of WHY1, 3 or AtRNH1C suppressed RNApol binding. In contrast, WHY1 and 3 promoted recruitment of PEP RNApol	*A. thaliana*	WHY1 WHY3	Wang et al. (2021)

## 2 THE WHY PROTEIN FAMILY

**FIGURE 1:**
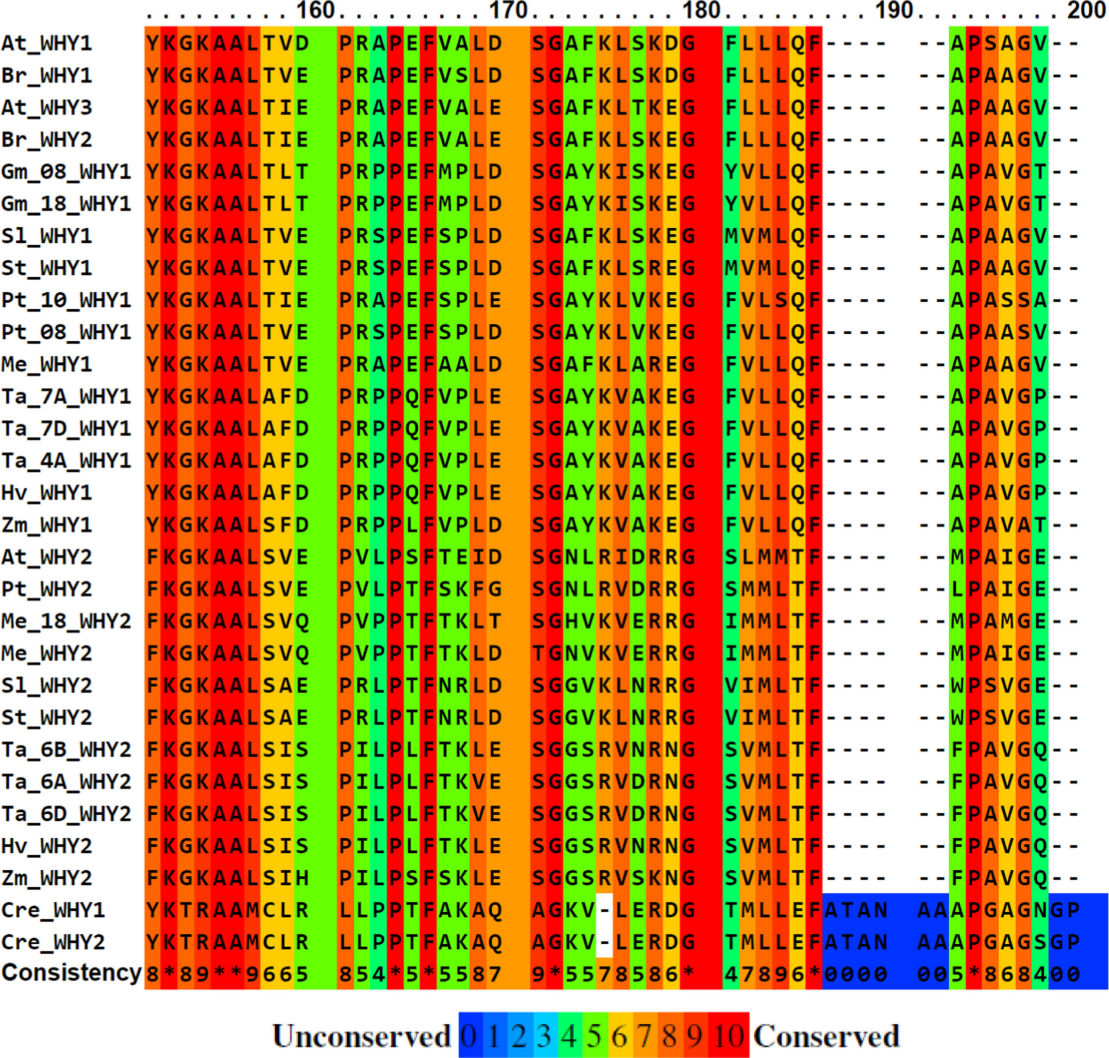
Sequence alignments of WHIRLY genes in a range of species. The Profile ALIgNmEnt (PRALINE) multiple sequence alignment toolkit with homology‐extended alignment illustrates the conserved KGKAAL DNA binding domain in WHIRLY proteins (Bawono & Heringa, 2014; Simossis & Heringa, 2005). PRALINE allows quick visual comparisons of the best characterized species used in WHIRLY research, or which published sequence data are available. Species are as follows: *Arabidopsis thaliana* (At), *Brassica rapa* (Br), *Glycine max* (Gm), *Hordeum vulgare* (Hv), *Manihot esculenta* (Me), *Populus trichocarpa* (Pt), *Oryza sativa* (Os), *Sorghum bicolor* (Sb), *Solanum lycospericum* (Sl), *Solanum tuberosum* (St), *Triticum aestivum* (Ta), *Zea mays* (Zm), *Chlamydomonas reinhardtii* (Cre). The scoring scheme works from 0 for the least conserved alignment position, up to 10 for the most conserved alignment position. The colour‐coded assignments are scored as conservation of alignment position from unconserved (blue) to highly conserved (red)

The small family of WHY proteins have a characteristic “whirligig” secondary structure and a conserved KGKAAL DNA binding domain that is found largely in angiosperms (Figure 1). WHY proteins are highly conserved in seed plants, and they are even present in some plastid‐containing algae (Lee et al., 2016). WHY proteins are essential in recognition and processing of ssDNA, stabilizing the ssDNA intermediates during DNA replication, recombination and repair, telomere maintenance and regulation of gene expression. WHIRLY domains are comprised of four structural topologies that are approximately 180‐amino‐acid‐long and characterized by two anti‐parallel four‐stranded β sheets stabilized by a C‐terminal helix‐loop‐helix motif (Cappadocia et al., 2013; Desveaux et al., 2005). WHY proteins form tetramers (Desveaux et al., 2002) and further assemble into hexamers of the tetramers, that is, 24‐mers (Cappadocia et al., 2012).

WHY was first identified as a factor, named p24, that bound to the inverted repeat sequence of the elicitor response element (ERE) on the promoter of the potato pathogenesis‐related (PR)‐*10a* gene, acting as a transcriptional activator in response to infection by the oomycete pathogen, *Phytophthora infestans* (Despres et al., 1995; Desveaux et al., 2000). Most plant species have two WHY proteins, WHY1 and WHY2. WHY1 is a nuclear‐localized protein that also targets to chloroplasts (Grabowski et al., 2008). WHY2 is targeted to mitochondria but there is evidence of potential localization toplastids and nuclei (Huang et al., 2020; Krause et al., 2005). A third WHY protein, which is targeted to chloroplasts, has been found only in Arabidopsis (Krause et al., 2005). WHY1 appears to interact with WHY3 synergistically in plastids to maintain organelle genome stability and metabolism (Guan et al., 2018; Maréchal et al., 2008). However, the situation with regard to the localization of the WHY proteins may be more complicated because in organello protein transport experiments using intact mitochondria and chloroplasts suggested that WHY3 can be dually targeted to chloroplasts and mitochondria (Golin et al., 2020). Previous analysis of in vivo targeting and in vitro organellar transport studies presented conflicting results depending on the assay used to determine WHY protein subcellular localization (Krause et al., 2005). Thus, there remains some uncertainty on how the different WHY proteins partition between organelles in planta and the extent to which this varies on growth stage or under stress.

**FIGURE 2:**
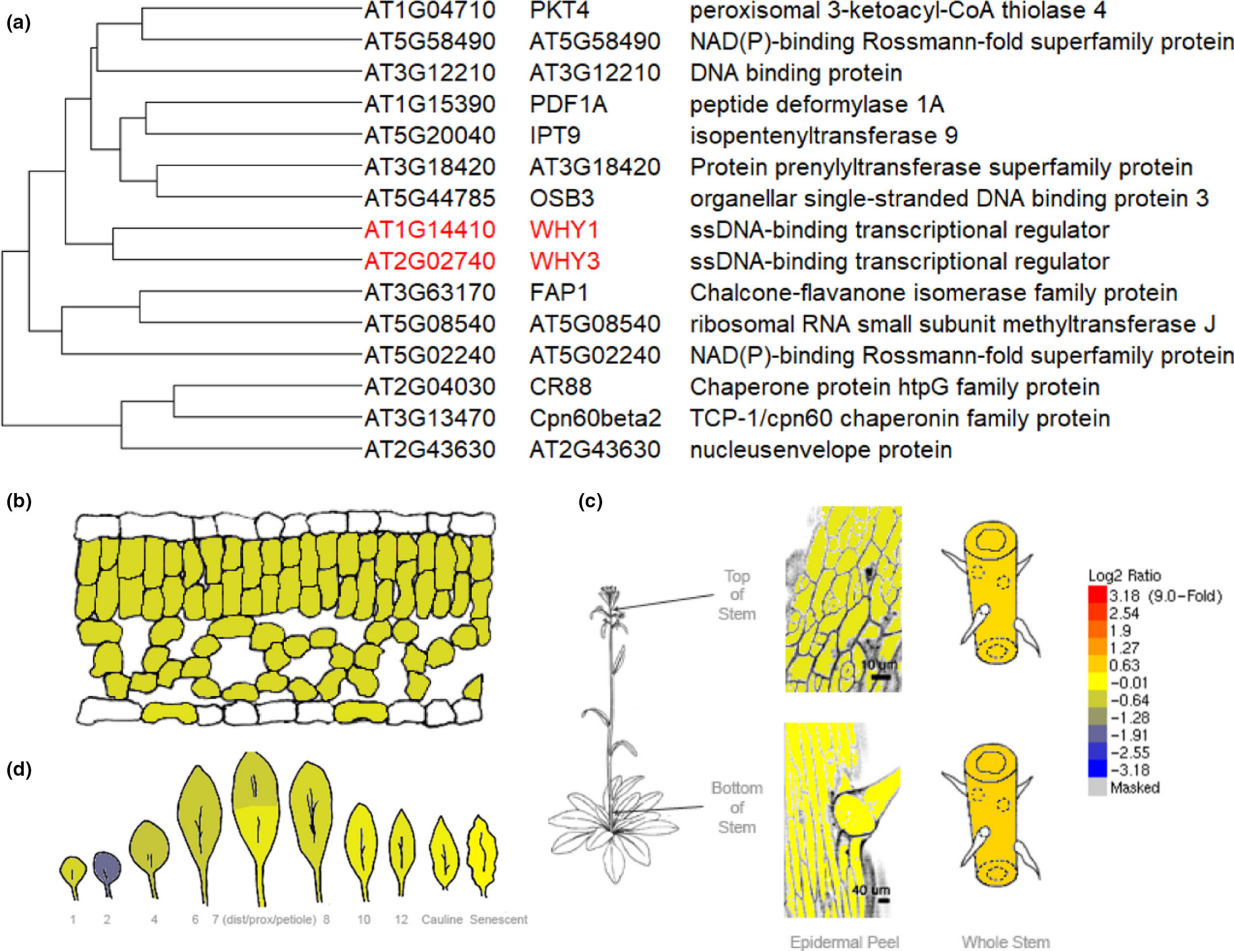
A summary of *WHY1* and *WHY3* expression obtained by analysis of published transcriptome data. Co‐expression of *WHY1* (*AT1G14410*) and *WHY3* (*AT2G02740*). (a) Identification of genes with similar expression patterns to *WHY1* at https://www.michalopoulos.net/act/. Gene ontology analysis of the 119 genes with most similar expression pattern identified significant enrichment in genes encoding plastid localized proteins (adjusted *p* value 5.9 × 10^−53^). (b–d) Comparative expression of *WHY1*and *WHY3* identified no significant differential expression between tissues (fold difference < ±2) (b) leaf mesophyll and guard cells (c) stem and epidermis of shoot tissue (d) rosette leaves. Tissue types and normalization of expression values is described by Winter et al. (2007)

A comparison of the amino acid sequences of the potato p24 to Arabidopsis WHY homologs showed the highest similarity (68%) to AtWHY1 protein, followed by AtWHY2 (42%) and then AtWHY3 (30%) (Krause et al., 2005). Furthermore, plastid‐localized AtWHY1 and AtWHY3 proteins have 77% sequence similarity to each other (Desveaux et al., 2005). A higher degree of homology was found between WHY proteins in monocots compared to dicots (Desveaux et al., 2005). There is therefore considerable potential for functional redundancy between the plastid‐localized proteins. This redundancy may explain the absence of marked phenotypes in some single and double mutants, or alternatively the functions of both WHY proteins may be required only under certain growth conditions. However, relatively little is known about the spatio‐temporal and environmental responsive expression patterns of the different *WHY* genes (Figure 2), and hence the importance of each from may only become apparent under different environmental or developmental conditions. Analysis of published transcriptomic data is consistent with a high degree of *WHY1* and *WHY3* co‐expression across a range of growth stages and treatments (Figure 2). These genes displayed similar expression patterns to genes encoding plastid localized proteins (FDR 5.9 × 10^−53^) (Zogopoulos et al., 2021). High expression in dividing tissues is consistent with roles for both WHY1 and WHY3 in plastid DNA metabolism (Winter et al., 2007).

Genes with a high degree of homology to WHY1 have been reported in unicellular green algae such as *Chlamydomonas reinhardtii* and *Klebsormidium flaccidum*, as well as in common liverwort *Marchantia polymorpha* (Krupinska et al., 2014 and Kobayashi et al., 2015). However, no homologous genes were reported in the cyanobacterium *Synechococcus elongates* or in *Ceratocystis paradoxa* and *Cyanidioschyzon merolae* (Kobayashi et al., 2015). Together, these results suggest that duplication of the original *WHY* gene may have occurred as an early eukaryotic component of chloroplast nucleoids.

**FIGURE 3:**
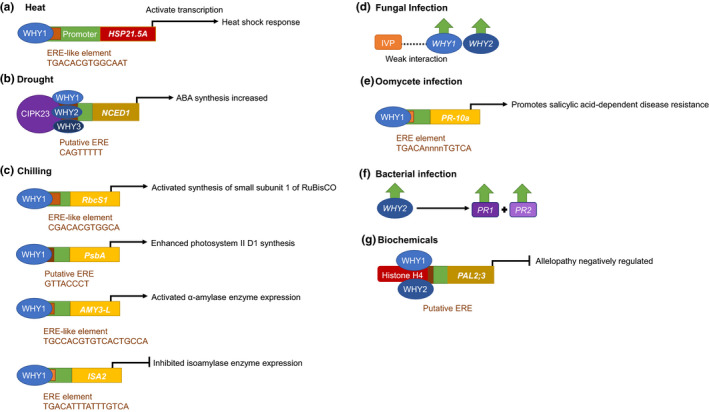
WHY1‐mediated regulation of nuclear gene expression. (a) WHY1 binds to the ERE‐like element in the promoter of the *HSP21*.*5A* gene of *Solanum lycospericum* (tomato) in response to heat stress to activate the heat shock response (Zhuang, Gao, et al., 2020); (b) WHY proteins bind to an upstream region in the *NCED1* gene promoter and to the CIPK23 promoter of *Manihot esculenta* (cassava) in response to drought stress to activate abscisic acid biosynthesis (Yan, Ning, et al., 2020); (c) WHY1 binds to the ERE‐like element in the *RbcS1* promoter in response to chilling stress. WHY1 is also required for the activation of the synthesis of the D1 protein of photosystem II (Zhuang, Wang, et al., 2020). WHY1 binding to the ERE‐like element of the *AMY3*‐*L* promoter to activate α‐amylase, which is required for starch degradation, and to the ERE element of the *ISA2* promoter to inhibit isoamylase‐mediated starch‐synthesis (Zhuang, Gao, et al., 2020). WHIRLY proteins are upregulated upon infection of pathogens and interact in biotic defence pathways. d) WHY1 is upregulated upon fungal infection by *Ustiligo maydis* in *Zea mays* (Kretschmer et al., 2017) and WHY1 was found to have a weak interaction with the Irregular Vascular Patterning (IVP) in *Cucumis sativus*, which functions in *Botrytis cinerea* resistance. Furthermore WHY2 was upregulated by *B. cinerea* infection in *Solanum lycospericum* (Akbudak & Feliz, 2019). Both WHY1 and WHY2 were upregulated in *Fragaria* × *ananassa* cv. Primoris upon *Colletotrichum acutatum* infection (Higuera et al., 2019). WHY proteins have also featured in oomycete infection where e) in addition to WHY1 upregulation upon infection by *Hyaloperonospora parasitica* (Desveaux et al., 2004), WHY1 was first discovered to act as a trans‐acting factor on Pathogenesis Related (PR) gene expression *Phytophthora infestans* where it binds to an elicitor‐response element (ERE) in the promoter of the PR‐10a gene which acts on the salicylic acid‐dependent disease resistance pathway (Despres et al., 1995; Desveaux et al., 2000). During f) bacterial infection, WHY2 has been found to be upregulated upon infection by *Ralstonia solanacearum* (previously *Pseudomonas solanacearum*) in *S. lycospericum*, which induced the transcriptional expression of PR1 and PR2 defence‐related genes (Zhao et al., 2018). More recently, WHY proteins have also been found to work in allelopathy defence against g) biochemical produced by competitive plants in allelopathy. Upon release of allelochemicals by neighbouring plants WHY1 and WHY2 were bound with Histone H4 (all of which were upregulated) to the promoter of PAL2;3, potentially to an ERE element, in Oryza sativa to negatively regulate allelopathy (Fang et al., 2020)

A growing body of evidence demonstrates that WHY proteins function as transcriptional activators of a large number of nuclear genes, particularly in response to environmental stress (Figure 3). For example, WHY1 binds to the AT‐rich region of kinesin gene promoter to activate kinesin gene expression (Xiong et al., 2009). Moreover, the barley WHY1 protein binds to the GTCAAT motif of *S40* promoter (Krupinska et al., 2014) and to a combination GTNNNAAT motif and an AT‐rich motif of downstream target genes in Arabidopsis, such as *WRKY53*, *WRKY33*, *SPO11* and *PR1* to regulate leaf senescence and related processes (Huang et al., 2018; Miao et al., 2013; Ren et al., 2017). WHY1 also influences telomere maintenance (Yoo et al., 2007) and microRNA synthesis (Świda‐Barteczka et al., 2018). Plants with reduced abundance of WHY1 show delays in chloroplast development (Krupinska et al., 2019) and leaf senescence (Kucharewicz et al., 2017), with altered responses to low nitrogen availability (Comadira et al., 2015) and exposure to continuous high light (Świda‐Barteczka et al., 2018). There are few reports of the phenotypes of plants overexpressing WHY1 or WHY3 (Ren et al., 2017), but transgenic tomato lines overexpressing SlWHY1 showed enhanced resistance to chilling stress through altered photosynthetic gene expression and modified starch accumulation (Zhuang, Wang, et al., 2020).

Arabidopsis plants over‐expressing WHY2 were smaller than the wild type with dark‐green distorted leaves, exhibiting early senescence with shorter siliques and fewer seeds than the wild‐type plants (Maréchal et al., 2008). WHY2 plays a key role in the maintenance of integrity of mitochondrial DNA and has also been implicated in protecting mitochondrial DNA from degradation during pollen development (Cai et al., 2015). While the *why2*‐*1* mutants do not show any obvious difference in the phenotype compared to the wild type (Maréchal et al., 2008), a major proportion of the leaf mitochondria have an aberrant structure that is characterized by disorganized nucleoids, fewer cristae, and a low matrix density (Golin et al., 2020). It is possible that these structural phenotypes may lead to growth defects under specific stress conditions. In the mitochondria, WHY2 binds to DNA and RNA, activating the expression of the *NAD1* and *ccb382* genes. Transgenic potato lines that are deficient in WHY2 had lower levels of mitochondrial DNA repair and recombination transcripts, together with a higher accumulation of reactive oxygen species (ROS) than the wild type (Meng et al., 2020). In the nuclei, WHY2 binds to the promotors of the *SWEET11*/*15* genes that encode sucrose transporters. It also increases the expression of genes involved in jasmonate signalling and associated defence responses. Thus, WHY2 has the potential to influence carbon re‐allocation between organelles and the nucleus (Huang et al., 2020).

## 3 PARTITIONING OF WHY PROTEINS BETWEEN ORGANELLES AND THE NUCLEUS

The compartmentation of the WHY proteins is fundamentally important to their functions in plant growth, development and defence. The WHY proteins are synthesized in the cytosol and delivered to the mitochondria and chloroplasts compartments via their targeting signals. However, increasing evidence suggests that the complement of WHY1, WHY2 or WHY3 in any given compartment is not fixed and that the WHY proteins can move between compartments in response to metabolic or environmental triggers. The expression of *WHY3* is enhanced in the developing shoots of the *why2‐1* mutant, which may indicate that WHY3 can compensate to some extent for the lack of WHY2 or may reflect pleiotropic effects arising from loss of WHY2 (Golin et al., 2020). The possibility of multiple organellar targeting would complicate the interpretation of data concerning the characterization of single mutants lacking WHY1, WHY2 or WHY3, and even some double mutants such as the *Atwhy1why3* mutants. While the mechanisms and processes that mediate such relocation events are largely uncharacterized, post‐translational modifications (PTMs) appear to play a role in the control the compartmentation of the WHY proteins. Protein phosphorylation has been reported to influence the compartmentation of WHY1 between the plastids and nuclei (Ren et al., 2017). WHY1 was shown to undergo reversible PTMs, the phosphorylation of WHY1 being catalysed by Calcineurin B‐Like‐Interacting Protein Kinase 14 (CIPK14) through a yeast two‐hybrid screen (Ren et al., 2017). Arabidopsis plants overexpressing *CIPK14* showed increased accumulation of WHY1 in the nucleus and decreased accumulation of WHY1 in plastids. The nuclear localization of WHY1 was linked to delayed leaf senescence and a stay‐green phenotype (Ren et al., 2017).

The phosphorylation of AtWHY1 was shown to favour partitioning to the nucleus and decrease the abundance of AtWHY1 in chloroplasts, a process that was reported to increase with the age of the leaves (Guan et al., 2018). The ratio of WHY1 in the nucleus to WHY1 in the plastid was suggested to be determined by the level of CIPK14 expression. Presumably, the compartmentation of the WHY2 and WHY3 proteins is regulated in a similar manner but little is known about how such PTMs regulate the sorting of the WHY proteins under different conditions.

## 4 WHY PROTEIN FUNCTIONS IN PLASTID GENOME REPAIR MECHANISMS

**FIGURE 4:**
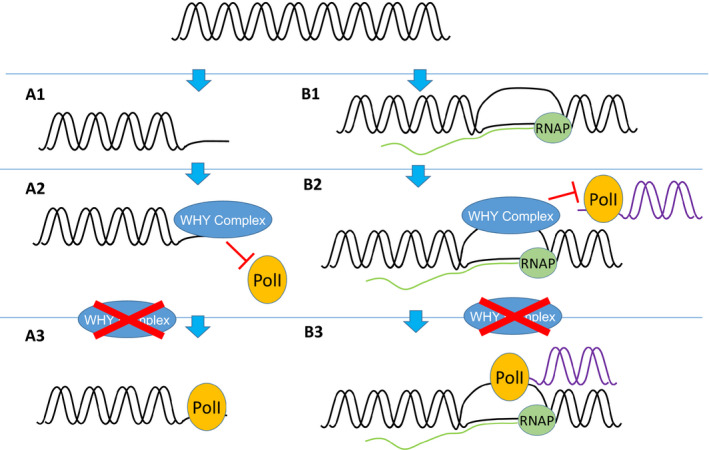
Model of WHIRLY involvement in DNA repair pathways. WHIRLY is involved in two pathways; (a1) by blocking microhomology‐mediated end joining (MMEJ) by DNA polymerases in the presence of AtWHY2 where single‐stranded regions are longer than 12 nucleotides (García‐Medel et al., 2019) and (b1) the accumulation of RNA: DNA hybrids (Pérez Di Giorgio et al., 2019). The transcribed DNA strand (black) is associated with the RNA (green) and RNA polymerase (RNAP). WHY proteins protect cp‐ and mtDNA by preventing the error‐prone MMBIR pathway in both chloroplasts and mitochondria (Cappadocia et al., 2010). This leads to the formation of (a2) error‐free HR, or (b2) R loops. In the absence of WHY proteins the double strand break could be repaired by MMEJ/ microhomology‐mediated break‐induced replication (MMBIR) by the recruitment of PolII (a1) alone or (b3) in conjunction with RNA, which are both error‐prone pathways

The putative DNA‐binding domain, KGKAAL (Figure 1), that is characteristic of WHY proteins (Krause et al., 2005) is essential for DNA binding but not tetramerization (Grabowski et al., 2008; Krause et al., 2005). This function is inherent to the role of WHY proteins in DNA repair mechanisms, as illustrated in Figure 4. In the leaves of monocotyledonous species such as barley, which show a developmental gradient from base to tip, *WHY1* is highly expressed in the meristematic region at the leaf base, with much lower expression levels found in the middle of the leaf or tip regions (Karpinska et al., 2022). The WHY1 protein is localized in the nuclei (60%) and proplastids (40%) of the cells at the base al the leaf (Karpinska et al., 2022). This finding suggests that *WHY1* plays an important function in genome stability during DNA replication in the proliferating cells in meristems as well as in vegetative cells.

**FIGURE 5:**
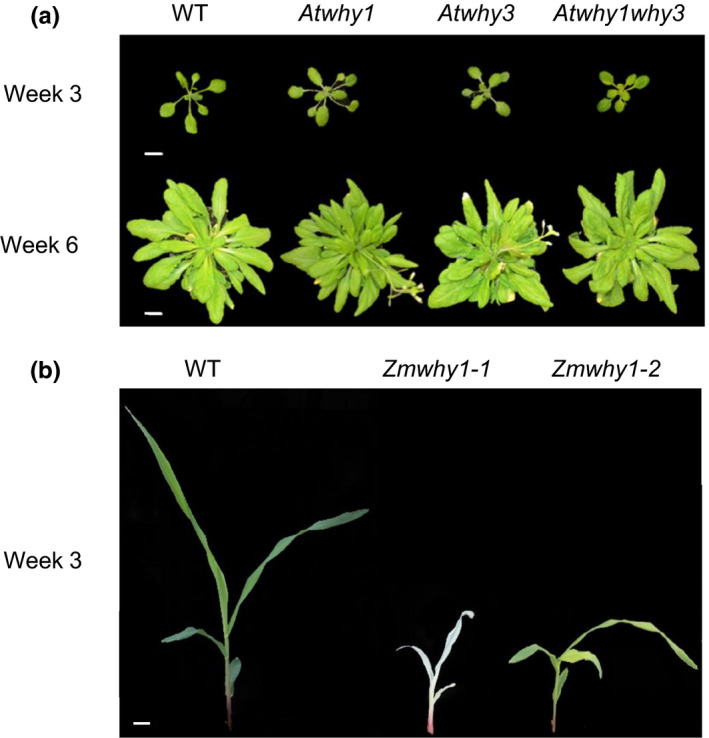
The phenotypes of *why* mutants of (a) Arabidopsis and (b) maize (*Zea mays*). (a) Rosette phenotypes of the three *Atwhy* mutants compared to the wild type at 3 and 6 weeks post‐germination grown under a 12 h photoperiod. No phenotypic differences were observed in any of the *Atwhy* mutants relative to the wild type during early (3 weeks) or late development (6 weeks). Scale bars are 10 mm. Unpublished data from this laboratory group. (b) Phenotypes of 9‐day old Zm*why1* mutant seedlings. Left to right: homozygous Zm*why1*‐*1* albino‐ivory, heteroalleic complementation cross of pale yellow *Zmwhy1*‐*1 and* Zm*why1*‐*2*, homozygous Zm*why1*‐*2* pale green phenotype and green wild type

Much of our current understanding of WHY protein functions in DNA repair comes from the characterization of mutants such as the *Atwhy1* and *Atwhy3* mutants (Figure 5). These mutants are phenotypically similar to wild‐type plants under standard growth conditions. The *Atwhy1why3* mutants are smaller than the wild type (Figure 5), with 4.6% of individuals showing variegated leaf sectors, suggesting that the plastid‐localized WHY proteins are involved with chloroplast development, from biogenesis to senescence (Maréchal et al., 2009). RNAi WHY1 knockdown barley lines are also phenotypically similar to the wild type, but the leaves have more chlorophyll and less sucrose than the wild type (Comadira et al., 2015). No differences in the photosynthesis rates of the mature leaves were observed (Comadira et al., 2015) but the sensitivity of photosynthesis to the stresses caused by nutrient limitation (Comadira et al., 2015) and high light (Świda‐Barteczka et al., 2018) was changed. Therefore, it appears that stress conditions reveal non‐redundant roles for WHY proteins. In contrast to observations in Arabidopsis and barley, the maize *Zmwhy1*‐*1* mutants were lacking in chlorophyll and not viable beyond the fourth leaf stage (Figure 5b; Prikryl et al., 2008). The *Zmwhy1*‐*2* plants were deficient in plastid ribosomes and with lower photosystem I and II activities, less cytochrome *b*6f complex and lower activities of ribulose‐1, 5‐bisphosphate carboxylase/oxygenase (Rubisco) (Prikryl et al., 2008). The reasons for these differences between the two monocotyledonous species, maize and barley, is not obvious but it may be related to differences in the modes of photosynthesis operating in the two species, maize leaves use the C4 photosynthesis pathway and have associated Kranz anatomy while the C3 photosynthesis pathway operates in barley and Arabidopsis.

The maintenance of organelle genome stability is crucial for chloroplast and mitochondrial functions, as well as plant growth and development. The mitochondrial and chloroplast genomes experience considerable homologous recombination (HR) and gene conversion events between comparable DNA sequences. The organellar DNA repair mechanisms thus play important roles in protecting genome stability. The WHY proteins promote plastid genome stability through binding to single‐stranded DNA and thereby suppressing the activity of specific recombination pathways (Maréchal et al., 2008, 2009). Like other organellar single‐strand binding proteins (OSBs), the WHIRLY family proteins are nuclear genome‐encoded. Such proteins are targeted to mitochondria (OSB1, OSB4 and WHY2), chloroplasts (OSB2, WHY1 and WHY3) or both the organelles (OSB3) through the anterograde signalling pathway (Mahapatra et al., 2021). WHY1, WHY2 and WHY3 are involved at the initial phase of HR in mitochondria and chloroplasts (Maréchal & Brisson, 2010).

Homologous recombination is a highly conserved pathway that promotes accurate repair using one of the multiple cpDNA copies present in the plastid (Wu et al., 2020) and has important roles in DNA replication (Zampini et al., 2015). HR activity is essential in plastids, as evidenced by the severe phenotype caused by disruption of the chloroplast localized HR recombinase, RECA1 (Shedge et al., 2007). Alternative recombination pathways are also active on organelles. Microhomology‐mediated break‐induced replication (MMBIR) is a form of recombination‐dependent replication present in plastids that only requires short (<30 bp) regions of homology (Maréchal et al., 2008, 2009), distinct to the longer regions used by HR. Loss of whirly in *Atwhy1why3* double mutants resulted in elevated levels of variegation that may result from cpDNA rearrangements through MMBIR (Maréchal et al., 2009). MMBIR may act as a backup pathway to HR, but has the potential to be highly mutagenic; repression of MMBIR by whirly proteins thereby maintains plastid genome stability (Maréchal et al., 2009). Reduction in RECA1 levels leads to a higher dependency on Whirly proteins to supress cpDNA recombination, likely due to important roles for RECA1 to prevent microhomology dependent rearrangements during cpDNA replication (Zampini et al., 2015). *Atreca1why1why3* mutants, where the *reca1* mutation is hypomorphic, display severe growth and fertility defects and visible tissue necrosis as well as a higher accumulation of plastid DNA rearrangements compared to the wild‐type plants (Duan et al., 2020). The *Atreca1why1why3* mutants accumulated ROS because of impaired photosynthetic functions, possibly due to defects in plastid gene expression resulting from severe plastid genome instability in these lines.

The bacterial recombinase RecA and its eukaryotic homologs of the Rad51 family plays a central role in HR. A recent report demonstrates that WHY1, WHY3 and RecA1 are associated with the chloroplast RNase H1 AtRNH1C protein and work together with to maintain chloroplast genome integrity (Wang et al., 2021). Accumulation of RNA: DNA hybrids which lead to displacement of the untranscribed DNA strand and are termed R‐loops and can result from high levels of transcription (Pérez Di Giorgio et al., 2019). Reducing the levels of transcription in plastids was able to alleviate the increased mutational load observed in *why1why3* mutants, providing evidence for whirly proteins in stabilizing R‐loops. Further evidence for a requirement for whirly in the presence of RNA: DNA hybrids was provided by *why1*/*3*/*Atrnh1c* triple mutants which showed more severe phenotypes than *Atrnh1c*, with highly bleached plants indicative of defective plastid function (Wang et al., 2021). Fluorescence microscopy and immunoprecipitation demonstrated that whirly proteins colocalize with AtRNH1C, in addition to RecA1, leading to the suggestion of a positive role of RNA: DNA hybrids in promoting HR (Wang et al., 2021).

Pol1B is required for chloroplast DNA replication. The *Atwhy1why3pol1b*‐*1* triple mutants exhibit a severe growth defect with yellow variegated leaves, lower photosynthesis rates and increased replication errors relative to the wild type (Lepage et al., 2013; Parent et al., 2011). An increase in replication stress resulting from POL1B deficiency is likely to result in the high dependency on whirly function to maintain plastid genome stability in the *pol1b* mutants (Zampini et al., 2015). The *Atwhy1why3pol1b*‐*1* leaves also accumulated ROS, presumably as a result of impaired photosynthetic electron transport functions (Lepage et al., 2013). Such increases may favour an increased susceptibility to programmed cell death (Redza‐Dutordoir & Averill‐Bates, 2016) and alter plastid to nucleus retrograde signalling. Since WHY2 may localize to plastids in some conditions (Huang et al., 2020), it follows that WHY2 could serve similar functions to the WHY1 and WHY3 proteins in these organelles. However, it is notable that the WHY2‐GFP used in this study with the native N‐terminus resulted solely in mitochondrial targeting (Huang et al., 2021).

Like the WHIRLY proteins, members of MutS mismatch repair family protein MSH1 are targeted to both chloroplast and mitochondria, where they play crucial roles in minimizing mutation rates in the organellar genomes (Virdi et al., 2016; Wu et al., 2020). The MutS homologue, MSH1 plays a role in the recognition of DNA damage associated with mismatches. The plant MSH1 contains a C‐terminal endonuclease domain, GIY‐YIG, which introduces DSBs and recruits the homologous DNA damage repair machinery. Of the duplicated MSH1, *MSH1A* and *MSH1B* genes in the moss *Physcomitrella patens*, the MSH1B protein interacts with RecA2 and RecG to maintain organellar genome stability (Odahara et al., 2009). Crucially, the MSH1 protein is localized in specialized sensory plastids in the epidermis, vascular parenchyma and meristems of higher plants. These “sensory” plastids participate in environmental stress sensing and trigger tissue‐specific signalling and systemic stress responses (Beltrán et al., 2018). The suppression of MSH1 triggers the expression of a wide range of stress and developmental pathways (Virdi et al., 2016). It is possible that like MSH1, WHY1 is localized in specialized plastids that play a key role in environmental sensing. These sensory plastids have overlapping but distinct protein profiles to chloroplast proteomes, and house many stress‐related proteins that are generally assigned to chloroplasts (Beltrán et al., 2018). However, many proteins that were identified in sensory plastids were not shared with mesophyll chloroplasts, implying that sensory plastids contain additional proteins that are involved in environmental sensing (Beltrán et al., 2018).

## 5 REGULATION OF PLANT DEVELOPMENT

The WHY proteins exert a number of important effects on plant development, firstly by acting as transcription factors in the nucleus that regulate the synthesis of hormones such as ABA and SA, and secondly in organelles, where they function as organizers of chloroplast and mitochondrial nucleoids. WHY1 deficient barley plants show delays in greening (Krupinska et al., 2019) and a delayed acquisition of full photosynthetic capacity (Comadira et al., 2015). These lines had a reduced chloroplast ribosome content and delayed activation of photosystem II, while showing an overaccumulation of light harvesting complex proteins with enhanced contents of chlorophyll b and xanthophylls. Such findings suggest that WHY1 is required for chloroplast biogenesis, consistent with roles in plastid genome maintenance or nuclear roles in coordinating expression of genes encoding plastid localized proteins (Krupinska et al., 2019). The finding that the potato WHY1 protein binds to the promoter region of the SlRbcS1 gene that encodes small subunit of ribulose‐1,5‐bisphosphate carboxylase/oxygenase (Rubisco) suggests that WHY proteins bind to a wide portfolio of nuclear genes (Zhuang, Wang, et al., 2020).

Loss of WHY1 in maize results in abnormal embryogenesis, lethality and albino embryos and seedlings, while some degree of albinism (Kretschmer et al., 2017), pale leaves (Figure 5) or variegation (Maréchal et al., 2009) has been reported in other species that are deficient in WHY proteins. Such variations in the reported phenotypes of WHY‐deficient plants are suggestive of significant differences in WHY protein functions in different species.

The seeds of Arabidopsis *why1* mutants were shown to have reduced sensitivity to salicylic acid (SA) and abscisic acid (ABA) during germination. Transgenic plants overexpressing the complete WHY1 sequence including the plastid targeting peptide (PTP) were hypersensitive to ABA while plants transformed to overexpress only the nuclear form of WHY1 and were insensitive to ABA (Isemer et al., 2012). This led the authors to conclude that WHY1 localized in the plastid mediated the responsiveness of seed germination to ABA. However, roles for WHY1 in ABA signalling in wild‐type plants remain to be elucidated.

The role of WHY1 as a transcription factor that regulates leaf senescence is well documented in the literature (Huang et al., 2018; Miao et al., 2013; Ren et al., 2017). RNAi‐mediated loss of WHY1 functions in barley (RNAi‐W1 lines) has effects on senescence and stress tolerance (Comadira et al., 2015; Kucharewicz et al., 2017). WHY1‐mediates regulation of senescence associated genes such as the barley *HvS40* gene (Janack et al., 2016). WHY1 may integrate the developmental regulation of senescence in response to biotic and abiotic stresses. For example, WHY1 deficiency had no effect on age‐ or dark‐dependent/low light senescence in barley leaves, whereas there was a clear effect on high light‐induced changes in the photosynthetic processes (Kucharewicz et al., 2017). The delay to senescence of RNAi‐W1 plants under high light led the authors to speculate that WHY1 has a role in light sensing and photooxidative stress (Kucharewicz et al., 2017).

Manipulation of WHY1 distribution between the nucleus and chloroplasts was shown to alter senescence and cellular redox homeostasis (Lin et al., 2019). The absence of WHY1 from the nucleus was linked to enhanced expression of the WRKY53 transcription factor, resulting in an early senescence phenotype (Lin et al., 2019). The accumulation of SA and the early senescence of the *why1* mutants could be prevented by ectopic expression of WHY1 in the nucleus (nWHY1). In contrast, expression of WHY1 in the plastids (pWHY1) enhanced SA accumulation. These authors showed that nWHY1 primarily controlled the expression of genes encoding proteins involved in plant development (Lin et al., 2020). WHY1 binds to the promoter region of the *isochorismate synthase1* (*ICS1*) activating expression. It also indirectly activates the expression of *S*‐*adenosyl*‐*l*‐*Met*‐*dependent methyltransferase1* (*BSMT1*) via ethylene response factor 109 (ERF109). In addition, WHY1 repressed the expression of *phenylalanine ammonia lyase* (*PAL1*) via R2R3‐MYB member 15 (MYB15) during the early stages of leaf development (Lin et al., 2020). The nWHY1 plants had a similar phenotype to the wild‐type phenotype, while the pWHY1 plants showed increased hydrogen peroxide accumulation and earlier leaf senescence. In these complementation experiments, however, these authors used constructs driven by the 35S promoter and hence the spatial regulation of these genes may differ from the native forms (Lin et al., 2020). Stay‐green phenotypes were reported in transgenic Arabidopsis lines overexpressing the *CIPK14* protein kinase, together with increased accumulation of WHY1 in the nuclei and decreased WHY1 levels in plastids, suggesting that CIPK14 may act as switch from plastid development to leaf senescence (Ren et al., 2017).

There are a number of senescence‐associated microRNAs in Arabidopsis leaves including microRNA840 (miR840), which appears only in plants of the genus Arabidopsis. Senescence is accelerated by miR840 via post‐transcriptional gene silencing of PPR and WHY3. This miRNA specifically targets the PPR and WHY3 genes in Arabidopsis. While PPR expression was mainly repressed on mRNA transcript level by cleavage, while WHY3 was predominantly translationally inhibited (Ren et al., 2022).

## 6 WHY1 AS A REGULATOR OF STRESS TOLERANCE

Many studies have reported increases in the levels of WHY transcripts in plants exposed to environmental stresses, such as salt and drought stress (Akbudak & Filiz, 2019; Yan et al., 2020), heat (Zhuang, Gao, et al., 2020), oxidative stress (Tunc‐Ozdemir et al., 2009) and infection with the fungus, *Botrytis cinera* (Akbudak & Filiz, 2019) (Figure 3). Similarly, the application of exogenous hydrogen peroxide favoured accumulation of WHY1 proteins in Arabidopsis chloroplasts (Lin et al., 2019). In contrast, citral, which is a naturally produced phytotoxic aroma‐compound in citric fruits, decreased the expression of all of the WHY genes (Graña et al., 2020). However, WHY2 transcripts were increased but not WHY1 transcripts in dehydrated chickpea seedlings (Lande et al., 2020).

Since the first report of WHY1 as a regulator of the expression of the *PR10*‐*a* gene in response to *P*. *infestans* attack in potato (Despres et al., 1995), WHY1 has been implicated in plant responses to biotic and abiotic stresses and shown to bind to the promoters of a wide variety of genes that encode proteins involved in stress tolerance, particularly those that contain ERF elements (Table 1; Figure 3). In many reports, the WHY1‐dependent changes in gene expression or other WHY1 interactions in the nuclei were linked to increased stress tolerance, as indicated in Table 1.

The ssDNA‐binding activity of AtWHY1 was induced upon infection by both avirulent and virulent pathogens (Desveaux et al., 2005). A Targeting Induced Local Lesions IN Genomes (TILLING) line with reduced AtWHY1 DNA‐binding activity showed enhanced susceptibility to infection by the oomycete pathogen *Peronospora parasitica* (Desveaux et al., 2005). A WHY1 overexpressing tomato line showed increased tolerance to the bacterial pathogen *Pseudomonas solanaceum* (Zhao et al., 2018), as well as increased drought tolerance (Figure 3). The maize *Zmwhy1*‐*1* and *Zmwhy1*‐*2* mutants (Figure 5) were more susceptible to infection with the pathogenic fungus, *Ustilago maydis* than the wild type (Kretschmer et al., 2017). However, only a weak interaction between *CsWHY1* and the SUPPRESSOR OF NONEXPRESSOR OF PATHOGENESIS‐RELATED GENES 1 (NPR1) (*CsSNI1*) was reported in cucumber plants infected with downy mildew (Yan, Ning, et al., 2020).

A recent study reported that the expression of *WHY2* exerted a strong influence over the expression of jasmonate signalling genes and associated defence responses (Huang et al., 2020). WHY2 was required for drought stress tolerance in potato. *SlWHY2* RNAi lines showed an enhanced wilting phenotype, as well as decreased fresh weight and chlorophyll contents, and decreased photosynthetic capacity (Meng et al., 2020). Similarly, WHY proteins were found to enhance drought tolerance in potato (Akbudak & Filiz, 2019). Epigenetic mechanisms involving modifications of the histone H3K9ac were reported for the WHY1‐dependent regulation of senescence and drought tolerance (Janack et al., 2016). The rice OsWHY1 and OsWHY2 proteins were found to bind to the promoter region of a phenylalanine ammonia‐lyase (PAL) gene, *OsPAL2*:*3* (Fang et al., 2019). OsMYB57_VP64_ and Kitaake lines were used to investigate the role of OsWHY and histone H4 in *OsPAL2*:*3* promoter regulation, two lines (Fang et al., 2019). These three DNA‐binding proteins were found to repress *OsPAL2*;*3* expression.

As discussed above, accumulating evidence demonstrates that WHY proteins function as transcriptional activators in the nucleus to co‐ordinate plant development and defence. The cassava WHY proteins bind to the PB element in the chloroplast‐localized *9*‐*cis epoxycarotenoid dioxygenase (NCED)1* gene, which activated expression and lead to increased levels of abscisic acid and drought tolerance (Yan, Liu, et al., 2020). WHY1 has been implicated in chilling stress resistance in potato, a process that was linked to the expression of the psbA gene that encodes the photosystem II (PSII) D1 protein and to regulation of the starch content in tomato (Zhuang, Wang, et al., 2020). The WHY1 protein in potato binds to the SlRbcS1 promoter increasing expression (Zhuang, Wang, et al., 2020). Photosynthetic CO_2_ assimilation rates were higher in three SlWHY1 overexpression lines and lower in three SlWHY1 RNAi lines compared with wild type (Zhuang, Wang, et al., 2020).

## 7 CONCLUSIONS AND PERSPECTIVES

**FIGURE 6:**
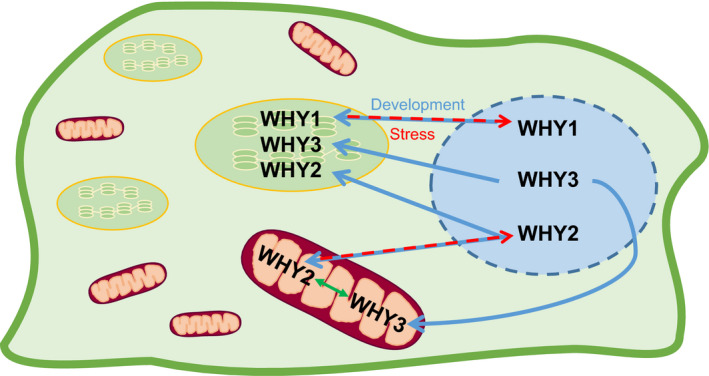
Schematic model of the intracellular localization of the WHIRLY proteins. The WHY proteins are targeted to organelles and are found in the nucleus (blue) at different stages of plant development. During growth WHY1 and WHY3 are targeted to the chloroplasts (green and yellow) while WHY2 is targeted to the mitochondria (maroon and orange). WHY3 and WHY1 have redundant functions (green double arrow). WHY2 has also been found in the chloroplasts (blue arrow). This model infers that WHY1 and WHY2 can be relocated to the nucleus in response to environmental stress (red dashed arrow)

Relatively little is known about the regulation of compartmentation, turnover and WHY proteins, but their wide presence throughout the plant kingdom strongly suggests conserved roles in plant physiology. As discussed above, there is a marked difference on the loss of WHY1 functions in different plant species. The absence of WHY1 is seedling lethal in maize, but results only in a delayed leaf greening in barley. The Arabidopsis *why1* and *why3* mutants have no visible phenotype, and only a very small percentage of *why1 why3* mutants show any phenotype. Hence, there is not only a difference in the nature of WHY1 functions between monocotyledonous and dicotyledonous species but there are also variations in WHY1 functions in different monocotyledonous species, and they probably also exist in dicotyledonous species. Nevertheless, accumulating evidence demonstrates that the WHY protein family fulfils a diverse range of important functions in plant development and stress tolerance. Many of these functions are related to their interactions with other proteins and ssDNA in chloroplasts, mitochondria and nuclei. The WHY family may thus be regarded as moonlighting proteins, that is, proteins that have two or more different functions, excluding those arising from gene fusion, homologous non‐identical proteins, splice variants, proteins with different PTMs, and those with a single function but active in different locations or on different substrates. They also belong to the increasing number of diverse proteins that can move from one compartment to another to fulfil different functions. As discussed above seed plants possess a minimum two WHIRLY proteins that can be localized in various intracellular compartments, as illustrated in Figure 6. WHY1 is localized in the chloroplasts and nucleus but the factors that regulate the partitioning of WHY1 between these intracellular compartments remains poorly understood. Senescence‐related changes in the phosphorylation state of the WHY1 protein is clearly important in targeting WHY1 to the nucleus in senescent leaves but little information is available about other situations, such as plant stress responses, where clearly WHY1 is localized in the nucleus to regulate the expression of defence response genes. Direct transfer of WHY1 from the plastids to the nuclei through contact sites or stromules (Hanson & Hines, 2018) is likely but remains to be proven. Moreover, the intracellular localization of WHY1 has a significant impact on the synthesis and responses to phytohormones such as ABA and SA that influence plant growth and defence, as evidenced by studies on seed germination and senescence (Isemer et al., 2012; Lin et al., 2020).

The WHY1 and WHY3 proteins appear to serve redundant functions under optimal growth conditions. However, such findings do not rule out the possibility that each form may serve specific functions in individual cell types or under stress conditions. While the degree to which WHY3 can functionally replace WHY2 during development remains to be established, it appears that WHY2 and WHY3 can relocate to different intracellular compartments upon the perception of appropriate signals and so serve overlapping functions in the mitochondria, plastids and nucleus. Such observations suggest that there is a degree of redundancy between the functions of all of the WHY proteins. The extent to which such relocation event occur may vary between species and so explain the discrepancies between phenotypes observed in *why* mutants between species. Much more work is required to understand how the intracellular compartmentation of the individual WHY proteins is managed under different developmental and environmental conditions. Similarly, loss of WHY protein functions will have direct and indirect effects on cell signalling. Accumulating evidence suggests that WHY1 acts a transcription factor in the nucleus. However, disruption of WHY functions also perturb organelle processes that can lead dramatic changes in the signalling networks associated with growth, development and stress responses.

The role of the WHY proteins in organelle to the nucleus retrograde signalling has long been suspected (Foyer et al., 2014) but the mechanisms involved remain to be established. We have previously examined the effects of inhibitors of chloroplast and mitochondrial functions on the relative abundance of *WHY1* and *WHY3* transcripts in 5‐day‐old Arabidopsis seedlings (Karpinska et al., 2017). Inhibitors such as lincomycin and norflurazon are frequently used to explore chloroplast to nucleus signalling while antimycin and salicylhydroxamic acid are used to characterize mitochondria to nucleus signalling. In contrast to the expression of photosynthesis‐associated nuclear genes (PhANGs), such as the gene encoding the light harvesting chlorophyll a/b binding complex B1 (LHCB1), which are decreased in the presence of the chloroplast inhibitors, the abundance of *WHY1* and *WHY3* transcripts was greatly increased in these conditions, as were the transcripts encoding the alternative mitochondrial terminal oxidase, ALTERNATIVE OXIDASE A1 (AOX1) of the mitochondrial electron transport chain. Moreover, the expression of *WHY1* and *WHY3* was greatly increased in the presence of mitochondrial inhibitors as was AOX1 (Karpinska et al., 2017). Such findings would suggest that are not regulated by the classic chloroplast to nucleus pathway but the expression of *WHY1* and *WHY3* may be regulated in response to mitochondrial signals. The expression of the AOX genes is commonly used as a marker for the mitochondrial retrograde regulation (MRR), which encompasses signals originating from mitochondria that induce nuclear transcriptional reprogramming (Ng et al., 2014). The WHY1, WHY2 and WHY3 are involved in maintaining structural integrity of mitochondrial and chloroplast genomes, and as such have roles in the signalling pathways that link organelle functions to nuclear gene expression. The activation and suppression of DDR in chloroplasts and mitochondria is inherently dependent on the transport of different proteins from the nucleus to the organelles. There is currently little information on how failure in DDR in chloroplasts and mitochondria is signalled to the nucleus. Similarly, there is a dearth of literature on the processes that regulate the degradation of WHY proteins, which also may have an impact on cell signalling. The molecular aspects of the WHY protein degradation, which may involve ubiquitination or other PTMS, are crucial to understanding how they function in retrograde signalling, particularly in relation to different developmental and or environmental conditions.

In summary, an increasing body of literature on WHY proteins demonstrate their importance in plant development and stress tolerance, not least because of their importance in the repair of organellar double‐strand breaks. Maintenance of organellar genome stability and the prevention of oxidative damage are major challenges for eukaryotic cells. The WHY proteins are important components of DNA damage repair mechanism in chloroplasts and mitochondria, which are central integrators of the metabolic, developmental and environmental cues that regulate plant development and stress signalling. As a central protein in organellar genome maintenance, the WHY family are under the coordinated control of the organelle and nuclear genomes. Like MSH1, WHY proteins are nuclear‐encoded, targeted to mitochondria and plastids and are universally present in plants. Both MSH1 and WHY1 are involved in organellar DNA binding and are thus nucleoid‐associated, and both serve functions far beyond their DNA‐binding roles, with nuclear transiting by WHY1 and epigenomic reprogramming by MSH1 (Mackenzie & Kundariya, 2020). The dual localization of proteins such as MSH1 and the WHY proteins is not only associated with important cellular processes in eukaryotic organisms but is also considered to be linked with multi‐functionalization and evolutionary advantage. Like MSH1, WHY1 is involved in the regulation of stress responses through direct and indirect effects on the expression of nuclear genes. As discussed above WHY1 also binds to telomeres and modifies telomere homeostasis, as well as participating in phytohormone signalling. Moreover, WHY1 represses the expression of *KP1*, which encodes a Kinesin‐like protein that functions in the mitochondria (Xiong et al., 2009), implying that plastid‐directed effects influencing WHY1 may alter mitochondrial regulation. Similarly, loss of WHY2 functions decreases photosynthesis and chlorophyll accumulation in chloroplasts (Meng et al., 2020).

We have previously speculated that WHY1 exists as an oligomer at the interface of the thylakoid membrane and nucleosome in the chloroplasts (Foyer et al., 2014) but it is the monomeric form that is involved during nuclear transit, particularly when the phosphorylated WHY1 protein is targeted to the nucleus (Ren et al., 2017). We speculated that conformational changes between the oligomer and the monomeric forms might be redox regulated (Foyer et al., 2014). More experiments are required to investigate the interactions between thioredoxin Z and the WHY1 and WHY3 proteins in chloroplasts to determine whether such interactions could regulate changes in the conformation or interactomes of the WHY1 and WHY3 proteins. Similarly, given the demonstrated roles of WHY1 in environmental responses, it will be interesting to determine whether WHY1 is localized in the sensory plastids that are located in the epidermis and vascular tissues (Beltrán et al., 2018). The intensive research efforts now being directed toward abiotic and biotic stress responses in plants may reveal further roles for the WHY proteins in stress sensing as in the array of robust organelle‐mediated defences.

We consider that WHY proteins are attractive targets for plant breeding strategies designed to produce plants that are better able to withstand environmental stress. Manipulation of *WHY* gene expression can clearly result is stay‐green phenotypes that are better able to withstand drought and cold stress. For example, genome editing technologies will allow precise modifications of each of the WHY proteins and their intracellular localizations in crop species to enhance stress tolerance and delay stress‐induced chlorosis. While a more comprehensive understanding of WHY protein homeostasis, intracellular compartmentation and functions, particularly in nuclei is clearly required, there is enormous potential for translation of this knowledge to improve sustainable agriculture, plant resistance mechanisms and associated plant breeding strategies. WHY proteins are crucial for organelle genome maintenance, and as such are under coordinated control of organelle and nuclear genomes. More research is required to shed new light on how WHY proteins participate in the signalling and pathways that control this co‐ordination.

## CONFLICT OF INTEREST

There are no conflicting interests.
